# ﻿Two new species of *Boletopsis* (Bankeraceae, Thelephorales) from Southwest China

**DOI:** 10.3897/mycokeys.89.83197

**Published:** 2022-05-04

**Authors:** Hong-Min Zhou, Qi Zhao, Qi Wang, Fang Wu, Yu-Cheng Dai

**Affiliations:** 1 Institute of Microbiology, School of Ecology and Nature Conservation, Beijing Forestry University, Beijing 100083, China Beijing Forestry University Beijing China; 2 Key Laboratory for Plant Diversity and Biogeography of East Asia, Kunming Institute of Botany, Chinese Academy of Sciences, Kunming 650201, China Kunming Institute of Botany, Chinese Academy of Sciences Kunming China; 3 Anqing Road 7 building 4-201, Fushun 113004, China Unaffiliated Fushun China

**Keywords:** Ectomycorrhizal fungi, phylogeny, taxonomy

## Abstract

Two new species of *Boletopsis*, *B.macrocarpa* and *B.tibetana*, are described and illustrated from Southwest (SW) China based on morphology, ecology and phylogenetic analyses by the internal transcribed spacer regions (ITS) and the large subunit of nuclear ribosomal RNA gene (nLSU). *Boletopsismacrocarpa* is characterized by big basidiocarps (up to 18 cm in diam), guttulate basidiospores, and the presence of gloeoplerous hyphae in context and growing in pure forest of *Pinusyunnanensis*. *Boletopsistibetana* is characterized by smaller pores (3–4 per mm), the presence of gloeoplerous hyphae in pileipellis, and the growth in forests of *Picea*. Phylogenetically, the two new species are grouped in two independent lineages nested in *Boletopsis*. In addition, one sample from Northeast China is temporarily treated as *Boletopsis* sp. 1 because of the single sample; another Chinese sample from SW China is sister to *B.grisea* in phylogeny, and it is treated as B.cf.grisea because the morphological difference between B.cf.grisea and *B.grisea* is indistinct. Furthermore, the main characteristics of *Boletopsis* species are listed, and a key to accepted species of *Boletopsis* is provided.

## ﻿Introduction

*Boletopsis* Fayod was established by Fayod based on *B.leucomelaena* (Pers.) Fayod originally described from Europe (Niemela and Saarenoksa 1989) and is characterized by annual basidiocarps with poroid hymenophore and central to lateral stipes, generative hyphae with clamp connections, and angular to tubercular, hyaline to pale brownish basidiospores which are negative in Melzer’s reagent (Ryvarden and Melo 2017). Previously, seven species, *B.grisea* (Peck) Bondartsev & Singer, *B.leucomelaena*, *B.mediterraneensis* G. Moreno et al., *B.watlingii* Blanco-Dios (=*B.perplexa* Watling & Jer. Milne, Nom. inval., Blanco-Dios 2018), *B.smithii* K.A. Harrison, *B.nothofagi* J.A. Cooper & P. Leonard and *B.atrata* Ryvarden, were accepted in the genus, and the first four species have a distribution in Europe (Ryvarden and Melo 2017), *B.grisea*, *B.watlingii* and *B.smithii* occur in North America (Watling and Milne 2008), and *B.nothofagi* and *B.atrata* were described from New Zealand and Thailand, respectively (Hjortstam and Ryvarden 1982; Cooper and Leonard 2012). Five of these seven species were analyzed by molecular techniques (Watling and Milne 2008; Cooper and Leonard 2012; Crous et al. 2019). In addition, *Boletopsissubsquamosa* (L.) Kotl. & Pouzar and *B.subcitrina* Corner were recorded in *Boletopsis* (Kotlába and Pouzar 1957; Corner 1989), but the former was considered as a synonym of *Albatrellusovinus* (Schaeff.) Kotl. & Pouzar (Donk 1974; Ryvarden and Gilbertson 1993), and the latter was treated as *Corneroporussubcitrinus* (Corner) T. Hatt. (Hattori 2001).

*Boletopsis* is the ectomycorrhizal fungal genus in the family Bankeraceae, phylogenetically, *Boletopsis* is sister to *Hydnellum* P. Karst. and *Sarcodon* Quél. ex P. Karst (Cooper and Leonard 2012; Mu et al. 2021; Crous et al. 2019). Morphologically, *Boletopsis* is distinguished from other genera in the family by poroid hymenophore.

Species of *Boletopsis* are edible mushrooms in SW China, and they are sold in the local markets as “black bear’s-paw fungi”, but their scientific names are unknown. During an investigation on forest macrofungi in China, sampling efforts of *Boletopsis* were especially focused on, and the ecology of these samples was recorded. The aim of this study is to clarify the species of *Boletopsis* in China and to expound phylogenetic relationships among members in the genus.

## ﻿Materials and methods

### ﻿Molecular phylogenetic studies

Eleven samples of *Boletopsis* were collected from Liaoning Province, Xizang Autonomous Region (Tibet) and Yunnan Province in China and deposited in the Herbarium of the Institute of Microbiology, Beijing Forestry University (BJFC). Potential host trees of *Boletopsis* spp. were observed from field trips. The macro-morphology was based on fresh and dried specimens. The color terms in descriptions followed Anonymous (1969) and Petersen (1996). Micro-morphology was studied at magnifications 1000×, using a Nikon Eclipse 80i microscope with phase contrast illumination. The Melzer’s reagent, Cotton Blue and 5% KOH were used in the study. Drawings were made with the aid of a drawing tube. In the text the following abbreviations were used: IKI = Melzer’s reagent, IKI– = non-dextrinoid and non-amyloid, KOH = 5% potassium hydroxide, CB = Cotton Blue, CB– = acyanophilous, L = mean basidiospores length (arithmetic average of all basidiospores), W = mean basidiospores width (arithmetic average of all basidiospores), Q = variation in the L/W ratios between the specimens studied, n = number of basidiospores measured from number of specimens.

### ﻿DNA extraction and amplification

A cetyltrimethyl ammonium bromide (CTAB) rapid plant genome extraction kit (Aidlab Biotechnologies Co. Ltd., Beijing, China) was used to extract DNA from dried specimens following the manufacturer’s instructions with some modifications (Chen et al. 2015, 2016). PCR reactions were performed in the 0.2 mL tubes, along with 1 µL DNA, 29 µL specified primers. The ITS primers pairs were ITS5 and ITS4 (White et al. 1990); the nLSU primers pairs was LR0R and LR7 (Vilgalys and Hester 1990). The optimal annealing temperature and cycles were generated as: an initial denaturation at 95 °C for 3 min, followed by 34 cycles at 94 °C for 40 s, annealing at 54 °C (sometimes at 56 °C) and extension at 72 °C for 1 min, and a final extension at 72 °C for 10 min. The PCR procedure for nLSU was: initial denaturation at 94 °C for 1 min, followed by 34 cycles of denaturation at 94 °C for 30 s, annealing at 50 °C for 1 min and extension at 72 °C for 1.5 min, and a final extension at 72 °C for 10 min.

### ﻿Phylogenetic analyses

Fifty-three sequences used in phylogenetic analyses are listed in Table 1, including 24 sequences generated by this study and another 29 downloaded from the National Center for Biotechnology Information (NCBI) which mainly adapted from Cooper and Leonard (2012) and Crous et al. (2019). *Sarcodonimbricatus* (L.) P. Karst. was used as outgroup (Crous et al. 2019).

**Table 1.** Information on the sequences used in this study.

Species	Sample	Location	Hosts	GenBank Accession No.	
				ITS	nLSU
*Boletopsisgrisea*	UPS F-120382	Sweden	*Pinussylvestris*	MN536751	MN535646
*Boletopsisgrisea*	UPS F-153996	Sweden	*Pinussylvestris*	MN536742	MN535641
*Boletopsisgrisea*	AB 16-09-113	France	*Abiesalba*	MN536743	–
*Boletopsisgrisea*	AB 17-09-52	France	*Abiesalba*	MN536744	–
*Boletopsisgrisea*	AH 42971	Spain	*Pinuspinea*	MN536747	MN535642
*Boletopsisgrisea*	AH 44091	Spain	*Pinuspinaster*	MN536748	MN535643
*Boletopsisgrisea*	Rec 227658	USA	*Tsugacanadensis*	EF457899	–
*Boletopsisgrisea*	Rec 227659	USA	*Pinussylvestris*	EF457902	–
**Boletopsiscf.grisea**	**Dai 23070**	**China**	*Pinus*, *Quercus*	**OL673003**	**OL672990**
*Boletopsisleucomelaena*	UPS F-173290	Sweden	*Piceaabies*	MN536739	MN535638
*Boletopsisleucomelaena*	UPS F-575617	Sweden	*Picea*, *Populus*	MN536740	MN535639
***Boletopsismacrocarpa***	**Dai 21780**	**China**	*Pinusyunnanensis*	**OL673004**	**OL672991**
***Boletopsismacrocarpa***	**Dai 22727**	**China**	*Pinusyunnanensis*	**OL673007**	**OL672994**
***Boletopsismacrocarpa***	**Dai 22728**	**China**	*Pinusyunnanensis*	**OL673005**	**OL672992**
***Boletopsismacrocarpa***	**Dai 22729**	**China**	*Pinusyunnanensis*	**OL673006**	**OL672993**
***Boletopsismacrocarpa***	**Dai 22748**	**China**	*Pinusyunnanensis*	**OL673008**	**OL672995**
***Boletopsismacrocarpa***	**Dai 23064**	**China**	*Pinusyunnanensis*	**OL673009**	**OL672996**
***Boletopsismacrocarpa***	**Dai 23065**	**China**	*Pinusyunnanensis*	**OL673010**	**OL672997**
*Boletopsismediterraneensis*	AB 06-10-343	France	*Cedrusatlantica*	MN536717	–
*Boletopsismediterraneensis*	AB 15-11-97	France	*Cedrusatlantica*	MN536736	–
*Boletopsismediterraneensis*	AH 44070	Spain	*Pinusnigra*	MN536724	MN535630
*Boletopsismediterraneensis*	AH 44080	Spain	*Pinus*	MN536723	MN535629
*Boletopsismediterraneensis*	FR 2016250	France	*Pinushalepensis*	MN536726	–
*Boletopsismediterraneensis*	ML 410112B	Cyprus	*Pinusnigra*	MN536719	–
*Boletopsisnothofagi*	PDD 96007	New Zealand	*Nothofagusfusca*	JQ417193	–
***Boletopsis* sp. 1**	**Dai 22172**	**China**	*Pinus*	**OL673011**	**OL672998**
***Boletopsistibetana***	**Dai 20896**	**China**	*Piceabalfouriana*	**OL673012**	**OL672999**
***Boletopsistibetana***	**Dai 20897**	**China**	*Piceabalfouriana*	**OL673013**	**OL673000**
*Boletopsiswatlingii*	Holden E150627 (E)	UK	*Pinussylvestris*	DQ408766	–
*Boletopsiswatlingii*	Wat. 28788 (E)	UK	*Pinussylvestris*	DQ408767	–
*Boletopsiswatlingii*	SMI 350	Canada	Unknown	FJ845401	–
***Sarcodonimbricatus***	**Dai 20314**	**China**	Unknown	**OL676807**	**OL678542**
*Sarcodonimbricatus*	NIFoS 1676	–	Unknown	MF421106	–

New sequences are shown in bold.

Raw chromatograms were aligned and edited using BioEdit Sequence Alignment Editor (Hall 1999), especially those chromatograms with double peaks at the start and the end of sequences. The ITS and nLSU sequences were aligned using MAFFT 7 online (https://mafft.cbrc.jp/alignment/server/), and applying the interative refinement method of G-INS-I (Katoh and Standley 2013). For aligned sequences, the ambiguous regions at the start and the end were deleted. Sequence alignment was deposited at TreeBASE (http://purl.org/phylo/treebase/; submission ID 29052).

The Maximum likelihood (ML) and Bayesian inference (BI) methods were used to conduct phylogenetic trees with ITS + nLSU matrix. The best-fit model was selected by ModelFinder (Kalyaanamoorthy et al. 2017), adopting Akaike information criterion (AIC). The model GTR + F + I + G4 was selected as the best-fit model for the ITS + nLSU matrix, lset nst = 6, rates = invgamma in Bayesian analysis. ML analysis was constructed by RaxmlGUI 1.2 (Stamatakis 2006). We performed default parameters in the ML analysis. A Bayesian tree was produced by MrBayes 3.1.2 (Ronquist and Huelsenbeck 2003) using the same model as ML analysis. Four Markov chains were run for 2 million generations for the analysis. Trees were sampled every 1000^th^ generation. The first 25% of sampled trees were discarded as burn-in, whereas others were used to construct a 50% majority consensus tree and for calculating Bayesian posterior probabilities (BPPs).

## ﻿Results

### ﻿Molecular phylogeny

**Figure 1.**
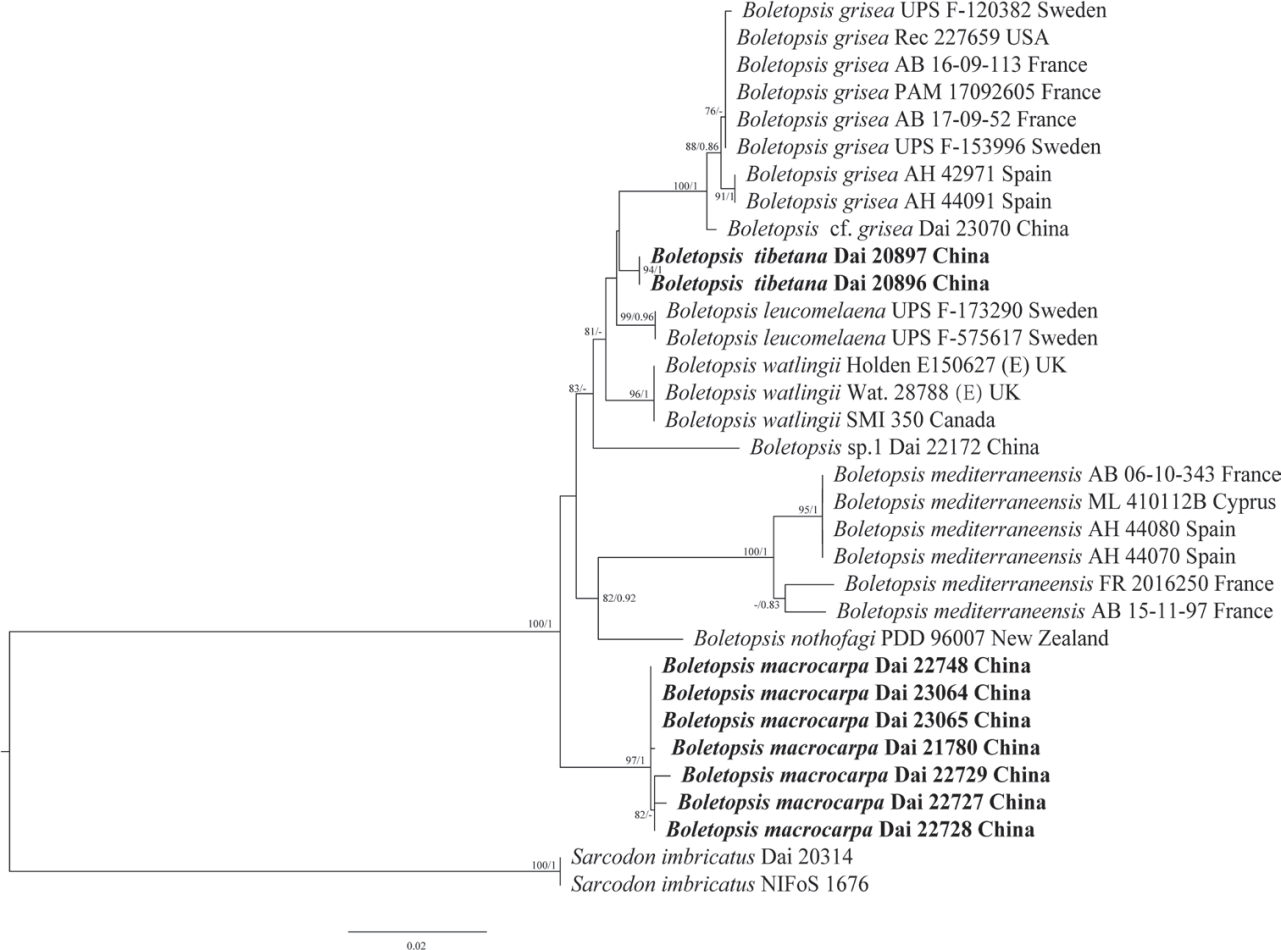
Phylogeny of species in *Boletopsis* generated by maximum likelihood based on ITS + nLSU sequence data. Branches are labeled with maximum likelihood bootstrap ≥ 75% and Bayesian posterior probabilities ≥ 0.80, respectively. New species are in bold.

A total of 33 ITS and 20 nLSU sequences were used in the phylogenetic analyses. The Bayes analysis and Maximum likelihood analysis resulted in a similar topology with an average standard deviation of split frequencies = 0.006494. All samples of *Boletopsis* form a monophyletic clade. Among the Chinese materials, the specimen Dai 23070 is sister to *B.grisea* samples from Europe and North America with a stable support (100/1). The specimen Dai 22172 has singleton position as a lineage, specimens Dai 20896 & 20897 and Dai 21780, 22727, 22728, 22729, 22748, 23064 & 23065 are grouped respectively in two lineages with high support (97/1, 94/1). So, two species are described from nine specimens grouped in two independent linages nested in *Boletopsis* clade, and specimens Dai 23070 and Dai 22172 are treated as Boletopsiscf.grisea and *Boletopsis* sp. 1, respectively (Fig. 1).

### ﻿Taxonomy

#### ﻿ Boletopsis macrocarpa

Taxon classificationFungiThelephoralesBankeraceae

Y.C. Dai, F. Wu & H.M. Zhou sp. nov.

7851BDF8-8E93-54A6-A363-AE4C8D473588

843792

Figs 2A , 3

##### Diagnosis.

Differs from other *Boletopsis* species by largest basidiocarps (up to 18 cm in diam) with grayish brown to dark gray upper surface, gloeoplerous hyphae present in context, guttulate basidiospores, and the fact that it grows in forests of *Pinusyunnanensis* at high altitude with open and slightly dry environments in SW China.

##### Holotype.

China, Yunnan Province, Nujiang, Lanping County, Xinshengqiao National Forest Park, on ground in forest of *Pinusyunnanensis*, alt. 3000 m, 2 September 2021, Dai 22728 (BJFC037301).

##### Etymology.

*Macrocarpa* (Lat.): referring to the species having largest basidiocarps.

##### Fruiting bodies.

Basidiocarps annual, terrestrial, centrally stipitate, solitary. Pilei circular or irregular, slightly depressed at center, with undulate and sharp margin, up to 18 cm in diam and 3 cm thick at center when fresh. Pileal surface grayish brown (5/6E4) with cream margin (4A2/3) when fresh, becoming blackish blue (20F8) to black upon drying, smooth, azonate. Pore surface white when fresh, becoming clay-buff (6D4) to fawn (7D/E4) upon drying; pores round to angular, some irregular, 1–3 per mm, mature pores bigger than juvenile ones; dissepiment thin, even to slightly lacerate. Context white when fresh, become pale mouse-gray (7C2) when dry, brittle, up to 2.5 cm thick when fresh. Tubes concolorous with pore surface, brittle, up to 5 mm long when fresh. Stipe pale ash-gray (19C2) when fresh, become mouse-gray (9F3) when dry, up to 6 cm long and 4 cm in diam when fresh.

**Figure 2.**
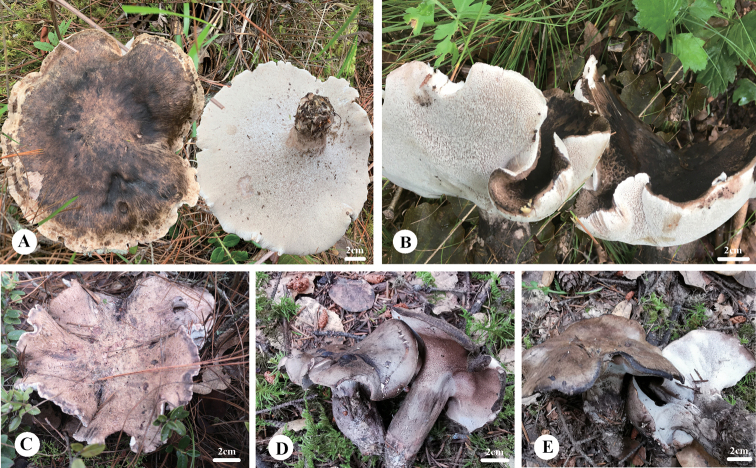
Basidiocarps of *Boletopsis* in China **A***B.macrocarpa* (Dai 22728) **B***B.* sp. 1 (Dai 22172) **C**B.cf.grisea (Dai 23070) **D–E***B.tibetana* (Dai 20896 and Dai 20897).

##### Hyphal structure.

Hyphal system monomitic; generative hyphae with clamp connections; gloeoplerous hyphae present, usually 3–11 μm in diam.

##### Pileipellis.

Pileipellis hyphae hyaline, thin- to thick-walled, 4–9 μm in diam; gloeoplerous hyphae rarely present; tissue darkening in KOH.

##### Context.

Contextual hyphae hyaline, thick-walled, rarely branched, interwoven, distinctly inflated, 5–25 μm in diam; gloeoplerous frequently hyphae present, thin-walled, reflective in Melzer’s reagent.

##### Stipitipellis.

Stipitipellis hyphae hyaline, usually thick-walled with a wide lumen, rarely branched, parallel along stipe, straight, uniform, 4–12 μm in diam; gloeoplerous hyphae rarely present.

##### Tubes.

Tramal hyphae hyaline, thin-walled, occasionally branched, interwoven, uniform, 2–4 μm in diam; gloeoplerous hyphae rarely present; cystidia and cystidioles absent; basidia clavate, tetrasterigmatic with a basal clamp connection, 14–19 × 6–7 μm.

##### Spores.

Basidiospores angular to tubercular with irregular ornaments, hyaline, thin-walled, with a guttule, IKI–, CB–, (4.5–)4.8–6(–6.2) × (3.7–)4–5 μm, L = 5.22 μm, W = 4.31 μm, Q = 1.20–1.22 (n = 90/3).

##### Additional specimens examined (paratypes).

China, Yunnan Province, Chuxiong, Wuding County, on ground in forest of *Pinusyunnanensis*, alt. 2400 m, 23 September 2021, Dai 23064 (BJFC037635), Dai 23065 (BJFC037636); Dali, Jianchuan County, Laojunshan Nature Reserve, on ground in forest of *Pinusyunnanensis*, alt. 3100 m, 29 August 2020, Dai 21780 (BJFC035681); Nujiang, Lanping County, Luoguqing Nature Reserve, on ground in forest of *Pinusyunnanensis*, alt. 3000 m, 3 September 2021, Dai 22748 (BJFC037321); Xinshengqiao National Forest Park, on ground in forest of *Pinusyunnanensis*, alt. 3000 m, 2 September 2021, Dai 22727 (BJFC037300), Dai 22729 (BJFC037302).

**Figure 3.**
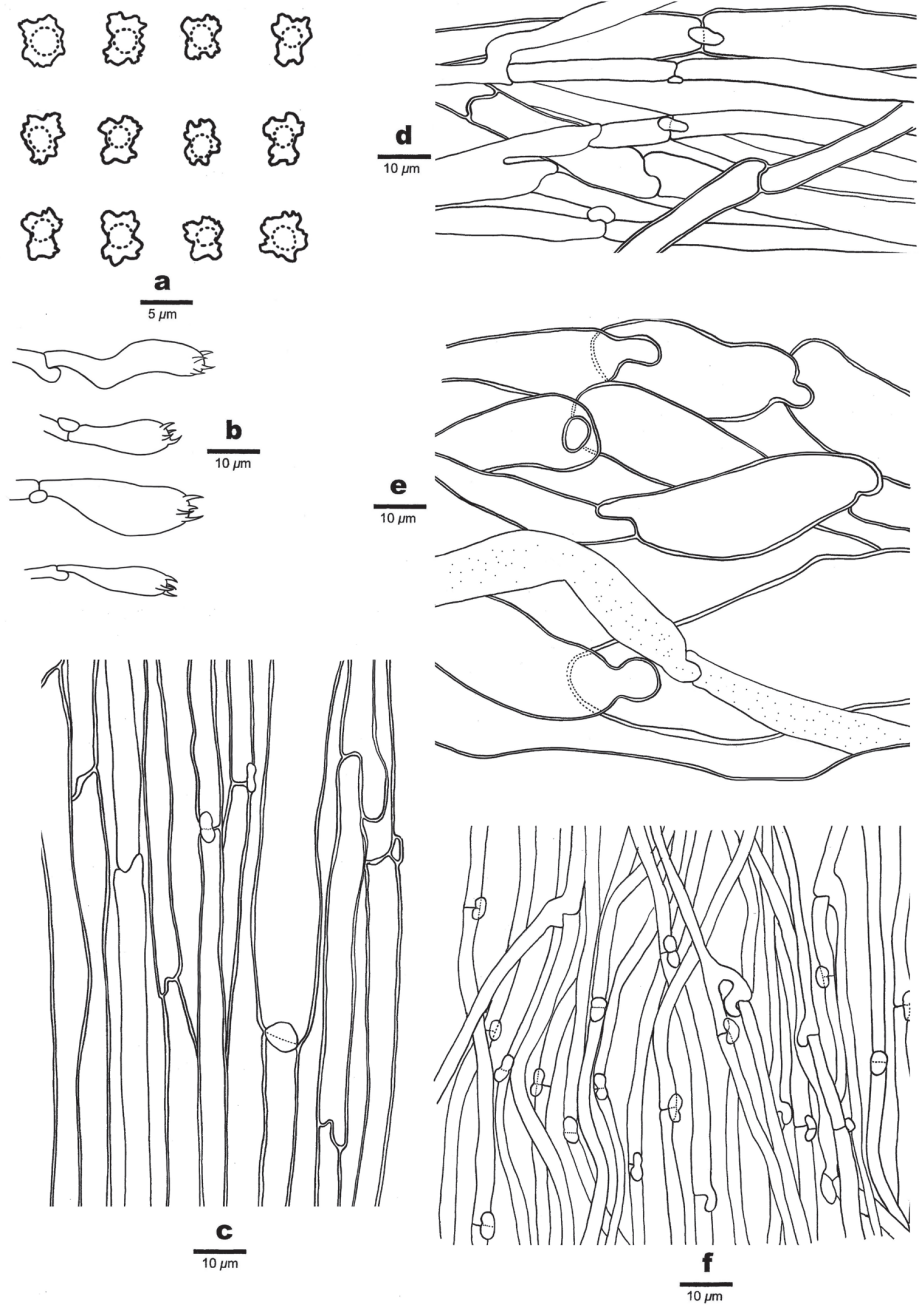
Microscopic structures of *Boletopsismacrocarpa* (Holotype) **a** basidiospores **b** basidia **c** stipitipellis hyphae **d** pileipellis hyphae **e** hyphae from context **f** hyphae from trama.

#### ﻿ Boletopsis tibetana

Taxon classificationFungiThelephoralesBankeraceae

Y.C. Dai, F. Wu & H.M. Zhou sp. nov.

1CC46588-7672-5BB7-8DBD-EFBC116ACABB

843793

Figs 2D–E , 4

##### Diagnosis.

Differs from other *Boletopsis* species by smaller pores (3–4 per mm), the presence of gloeoplerous hyphae in pileipellis and context, and the fact that it grows in the forest of *Picea* in Tibet, SW China.

##### Holotype.

China, Tibet, Linzhi, on ground in the forest of *Piceabalfouriana*, alt. 2900 m, 23 August 2019, Dai 20896 (BJFC032554).

##### Etymology.

*Tibetana* (Lat.): referring to the species having a distribution in Tibet.

##### Fruiting bodies.

Basidiocarps annual, terrestrial, centrally stipitate, solitary to confluent. Pilei convex, or irregular, with undulate and incurved margin, up to 7 cm in diam and 1 cm thick at center when fresh. Pileal surface vinaceous buff (4C4) to clay buff (6D4) when fresh, becoming mouse-gray (9F3) to black upon drying, smooth, azonate; margin concolorous with pileal surface. Pore surface white when fresh, become fawn (7D/E4) when bruised, ash-gray (19C2) when dry; pores round to angular, 3–4 per mm; dissepiment thin, entire to slightly lacerate. Context white when fresh, become ash gray (19C2) when dry, rigid, up to 9 mm thick when dry. Tubes concolorous with pore surface, brittle, up to 1 mm long when dry. Stipe concolorous with pileal surface, cylindrical or tapering to the base, up to 6 cm long and 2 cm in diam when fresh.

**Figure 4.**
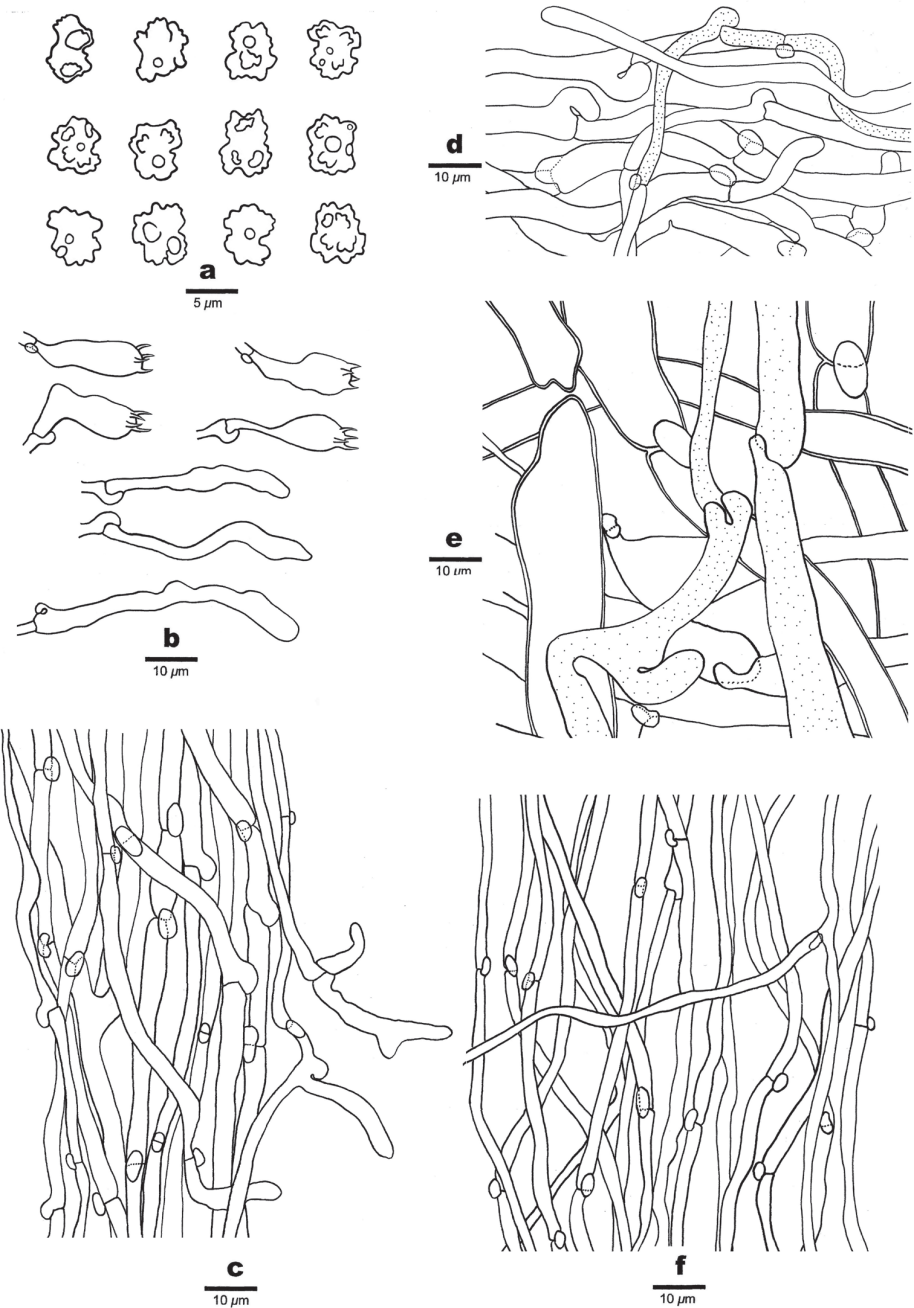
Microscopic structures of *Boletopsistibetana* (Holotype) **a** basidiospores **b** basidia and basidioles **c** stipitipellis hyphae **d** pileipellis hyphae **e** hyphae from context **f** hyphae from trama.

##### Hyphal structure.

Hyphal system monomitic; generative hyphae with clamp connections; gloeoplerous hyphae present, usually 3–11 μm in diam.

##### Pileipellis.

Pileipellis hyphae hyaline, thin-walled, with finger-shaped tips, 5–7 μm in diam; gloeoplerous hyphae frequently present, thin-walled, strongly reflective in Melzer’s reagent; tissue darkening in KOH.

##### Context.

Contextual hyphae hyaline, thick-walled, rarely branched, interwoven, distinctly inflated, 6–22 μm in diam; gloeoplerous hyphae present, thin-walled, strongly reflective in Melzer’s reagent.

##### Stipitipellis.

Stipitipellis hyphae hyaline, thin- to thick-walled, frequently branched, subparallel along stipe, straight, uniform, 2–6 μm in diam; gloeoplerous hyphae rarely present.

##### Tubes.

Tramal hyphae hyaline, thin-walled, occasionally branched, loosely interwoven, uniform, 2–4 μm in diam; gloeoplerous hyphae rarely present; cystidia and cystidioles absent; basidia clavate, tetrasterigmatic with a basal clamp connection, 13–25 × 6–8 μm; basidioles clavate, 22–40 × 3–4 μm.

##### Spores.

Basidiospores angular to tubercular with irregular ornaments, hyaline, thin-walled, IKI–, CB–, 5–6.5(–7) × 4–5(–5.2) μm, L = 5.55 μm, W = 4.41 μm, Q = 1.22–1.29 (n = 60/2).

##### Additional specimen examined (paratype).

China, Tibet, Linzhi, on ground in forest of *Piceabalfouriana*, alt. 2900 m, 23 August 2019, Dai 20897 (BJFC032555).

## ﻿Discussion

Previously seven species of *Boletopsis* were accepted mostly based on morphological examination, and five were confirmed by phylogenetic analyses (Cooper and Leonard 2012). In the present study, four distinct taxa of *Boletopsis* were found in China: *B.macrocarpa*, *B.tibetana*, B.cf.grisea and *B.* sp. 1 based on morphological and molecular evidence, and the phylogenetic relationship of seven *Boletopsis* taxa is analyzed (Fig. 1). The former two new species are proposed, but the latter two taxa require further collections and analyses.

Morphologically, *Boletopsismacrocarpa* and *B.mediterraneensis* share similar pileal surface, almost the same shape and size of basidiospores, and both species take *Pinus* as a potential host (Table 2), but the former has white fresh context which was unchanged when cut and hyaline basidiospores, while the latter has pale gray fresh context, becoming pale red when cut and hyaline to pale yellow-brown (Crous et al. 2019). In addition, *B.macrocarpa* has a distribution in SW China, while *B.mediterraneensis* is known in the Mediterranean area. *Boletopsisgrisea* resembles *B.macrocarpa* by almost the same size of pores and basidiospores, but the former has uniform grayish tinges for all upper surface, its gloeoplerous hyphae are present at pileipellis (Ryvarden and Melo 2017), while the upper surface is grayish brown to dark gray with cream margin and the gloeoplerous hyphae present in context in *B.macrocarpa* (Table 2).

**Table 2.** A comparison of morphology, ecology and distribution of *Boletopsis* species.

Species	Type Locality	Basidiocarps in diam (cm)	Pileal surface when fresh	gloeoplerous hyphae	Pores/mm	Basidiospores (μm)	Guttules in basidiospores	Hosts	Distribution	References
*B.atrata*	Thailand	2–5	black	–	2–3	4.5–6 in diam	–	*Quercus*, *Castanea*	Asia and North America	Hjortstam and Ryvarden 1982
*B.grisea*	Norway	5–18	gray–white to silvery gray, gray–brown, or brownish vinaceous	frequent in pileipellis	1–3	5–6.2 × 4–5	present	Pinaceae	Europe and North America	Watling and Milne 2008; Ryvarden and Melo 2017
*B.leucomelaena*	Norway	Up to 10	deep grayish to black	rarely present	1–3	5–6.5 × 4–5	present	mostly *Picea*	Europe	Ryvarden and Melo 2017
*B.macrocarpa*	China	12–18	grayish brown to dark gray	present in context	1–3	4.8–6 × 4–5	present	*Pinus*	Asia	This study
*B.mediterraneensis*	Spain	4–12	pale gray, brownish gray to ochraceous brown or dark brown	–	1–3	4.5–6.7 × 3.3–5.2	–	mostly *Pinus*, *Cedrus*	Europe	Crous et al. 2019
*B.nothofagi*	New Zealand	1–8	gray	present in context	2–3	5.3 × 4.1	–	*Nothofagus*	Oceania	Cooper and Leonard 2012
*B.smithii*	USA	4–5	dull orange	–	2–3	5.5–7 × 4.5–5.6	absent	–	North America	Harrison 1975
*B.tibetana*	China	5–7	vinaceous buff to clay buff	present in pileipellis	3–4	5–6.5 × 4–5	absent	*Picea*	Asia	This study
*B.watlingii*	UK	4–7	dark fuliginous brown to gray–brown	present in pileipellis	1–3	4.5–4.8 × 3.5–4.5	present	*Pinus*	Europe and North America	Watling and Milne 2006, 2008; Ryvarden and Melo 2017

*Boletopsistibetana* resembles *B.grisea* by almost the same shape and size of basidiospores. However, the latter species has bigger pores (1–3 per mm vs. 3–4 per mm, Table 2), and both species are phylogenetically distantly related. In fact, *Boletopsistibetana* has pores as 3–4 per mm, and other *Boletopsis* species have pores 1–3 per mm, so it is easily distinguished *B.tibetana* from other *Boletopsis* species.

Two species in *Boletopsis*, *B.atrata* and *B.smithii*, have so far no DNA data available, and their relationships with our new species are still unknown. Morphologically, *B.atrata* can be distinguished from our two new species by its small basidiocarps (2–5 cm in diam), verruculose basidiospores with regular ornaments (Hjortstam and Ryvarden 1982), while our new species have big basidiocarps (5–18 cm in diam), angular to tubercular basidiospores with irregular ornaments. *Boletopsissmithii* is different from our new species by its dull orange and smaller basidiocarps (4–5 cm in diam), and inflated hyphae (up to 17 μm in diam) in pileipellis and stipitipellis (Harrison 1975).

Although the specimen Dai 22172 forms an independent linage nested in *Boletopsis* clade in our phylogeny (Fig. 1), it is temporarily treated as *Boletopsis* sp. 1 because of the single sample. The taxon is characterized by the presence of scales at pileal margin, a bulbous stipe base, dentate pores, the presence of cystidioles, and the fact that it grows in a forest dominated by Pinussylvestrisvar.mongolica in NE China.

All European and North American samples of *Boletopsisgrisea* clustered together with a support (88/0.86), and a single Chinese sample Dai 23070 is sister to them (100/1). We treat the sample Dai 23070 as B.cf.grisea because no distinct morphological difference has been found between them to date. More samples and a multi-locus phylogeny are needed to clarify the status of the Chinese Boletopsiscf.grisea.

Species of *Boletopsis* form ectomycorrhizae with certain host plants, and the potential host trees may help to identify species, for instance, *Boletopsisleucomelaena* is usually associated with *Piceaabies* (L.) Karst. In Europe (Niemela and Saarenoksa 1989), and *B.nothofagi* are usually accompanied by *Nothofagus* in Oceania (Cooper and Leonard 2012). Almost all *Boletopsis* species are found in the Northern Hemisphere except *B.nothofagi*; most *Boletopsis* species grow coniferous trees in temperate areas and two species are known from more than one continent (Watling and Milne 2008; Ryvarden and Melo 2017). According to our field inventory, the two Chinese new species were found in temperate zone, and *Boletopsismacrocarpa* seems to prefer to pine forest at high altitude with open and slightly dry environments; *Boletopsistibetana* was found in coniferous forest dominant by spruce at high altitude with cold and humid environments. Previously numerous new species have been found in SW China (Dai et al. 2021; Wang et al. 2021), and the present paper confirms the fungal diversity is very rich in the montane forests of East Himalayas.

The main morphological characteristics, ecology and distribution of the accepted species of *Boletopsis* are summarized in Table 2.

### ﻿A key to accepted species of *Boletopsis* in the world

**Table:** 

1	Basidiospores verruculose with regular ornaments	***B.atrata***
–	Basidiospores oblong, angular to tubercular with irregular ornaments	**2**
2	Basidiospores < 5 μm long	***B.watlingii***
–	Basidiospores > 5 μm long	**3**
3	Pileal surface dull orange when fresh	***B.smithii***
–	Pileal surface vinaceous, grayish brown, dark gray or brownish to black when fresh	**4**
4	Pores 3–4 per mm	***B.tibetana***
–	Pores 1–3 per mm	**5**
5	Basidiospores oblong to tuberculate; associated to *Nothofagus* forest, distribution in Oceania	***B.nothofagi***
–	Basidiospores angular to tuberculate; associated to *Picea* or *Pinus* forest, distribution in Northern Hemisphere	**6**
6	Upper surface grayish brown with cream margin when fresh; distribution in Asia	***B.macrocarpa***
–	Upper surface brownish gray to blackish without cream margin when fresh; distribution in Europe and North America	**7**
7	Context pale gray, becoming pale red when cut	***B.mediterraneensis***
–	Context white, becoming darker when cut	**8**
8	Pileus dark gray to blackish, flesh brittle, usually associated to *Picea* forest	***B.leucomelaena***
–	Pileus grayish to grayish brown, flesh tough, usually associated to *Pinus* forest	***B.grisea***

## Supplementary Material

XML Treatment for Boletopsis macrocarpa

XML Treatment for Boletopsis tibetana
